# Vitreopapillary Traction Causing Optic Nerve Head Elevation

**DOI:** 10.1155/crop/3136288

**Published:** 2025-04-23

**Authors:** Mohamed M. Khodeiry, Mohammad Ayoubi, Christopher A. Dorizas, Carlos E. Mendoza-Santiesteban, Maja Kostic

**Affiliations:** ^1^University of Miami Health System, Bascom Palmer Eye Institute, Miami, Florida, USA; ^2^Department of Ophthalmology and Visual Sciences, University of Louisville, Louisville, Kentucky, USA; ^3^University of Miami School of Medicine, Miami, Florida, USA

**Keywords:** optic nerve head elevation, optic neuropathy, vitreopapillary traction

## Abstract

**Purpose:** The purpose of the study is to describe a case of vitreopapillary traction causing optic nerve head elevation.

**Observations:** This case report describes a 64-year-old male who presented with left cloudy vision for 3 days. Dilated fundus exam showed normal right optic nerve with glial tissue nasally and left optic nerve head elevation and peripapillary hemorrhages in the left eye. Magnetic resonance imaging of the brain and orbits, erythrocyte sedimentation rate, and C-reactive protein were normal. Optical coherence tomography showed bilateral dense vitreous adhesions to the optic disc nasally causing traction and optic nerve head elevation of the left eye. The patient was diagnosed with vitreopapillary traction causing optic nerve head elevation, and observation was recommended.

**Conclusions and Importance:** This case highlights the importance of clinical examination and ancillary testing in differentiating etiologies of optic disc elevation.

## 1. Introduction

Vitreopapillary traction (VPT) develops due to a fibrocellular proliferating membrane or either an anomalous or incomplete posterior vitreous detachment (PVD) excreting traction on the optic nerve head [1]. This has been described in patients with ocular pathologies such as diabetic retinopathy, central retinal vein occlusion (CRVO), and nonarteritic anterior ischemic optic neuropathy (NAION) [2]. The traction caused by the vitreous on the optic disc can lead to optic nerve head elevation, blurring of the disc margins, peripapillary hemorrhage, and possible disc leakage on fluorescein angiography [2]. Recent advancements in technology have allowed clinicians to detect and characterize VPT more accurately.

## 2. Case Report

A Caucasian 64-year-old male presented to the clinic with a complaint of blurred vision in the left eye that started 3 days prior to presentation. Past medical history was unremarkable. The patient had bilateral cataract surgery 4 years prior to presentation.

**Figure 1:**
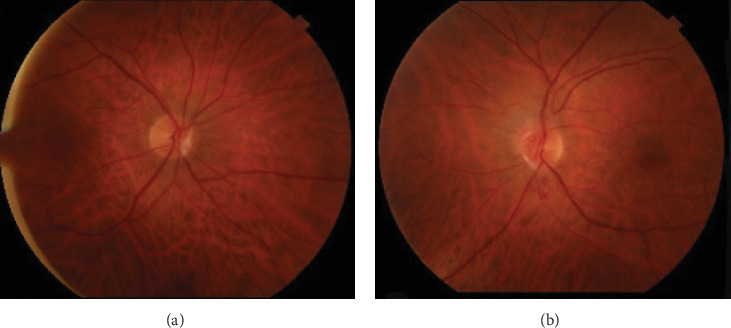
Fundus picture of both eyes showing glial tissue nasal to the optic disc of the right eye (a) and blurred optic disc margins and peripapillary hemorrhage of the left eye (b).

**Figure 2:**
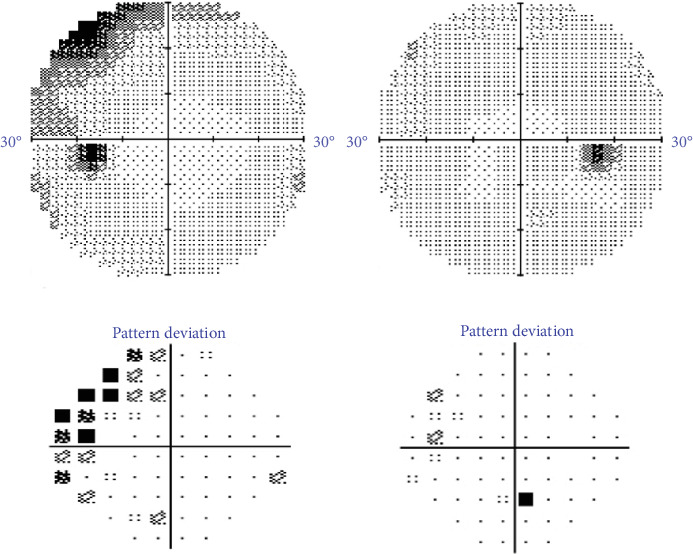
Humphrey 30-2 Swedish Interactive Testing Algorithm fast visual fields showing a full visual field of the right eye and superotemporal wedge-shaped scotoma in the left eye.

On ophthalmic exam, his best-corrected visual acuities were 20/20 in both eyes. Intraocular pressure was 13 mmHg in the right eye (OD) and 14 mmHg in the left eye (OS). Pupillary examinations, color vision, and ocular motility were normal. Anterior segment examination was unremarkable with no evidence of cells or flare in the anterior or posterior segment. On dilated fundus exam, a glial tissue nasal to the normal optic nerve head OD and optic nerve head elevation with peripapillary hemorrhages OS were noted (Figure 1a,b). The 30-2 automated visual field testing was normal OD and showed superotemporal wedge-like defect OS (Figure 2).

**Figure 3:**
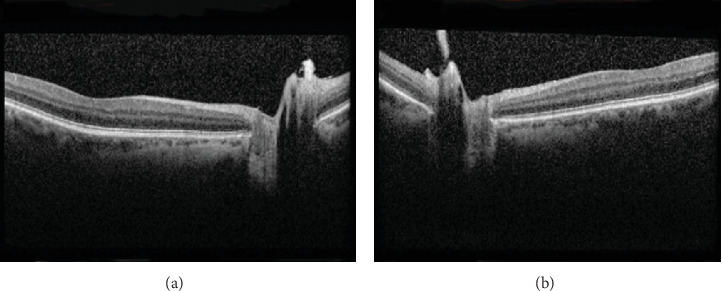
(a, b) Optical coherence tomography (OCT) of the optic nerve head showing vitreous traction on the optic discs of both eyes, with disc elevation of the left eye.

Optical coherence tomography (OCT) showed an average retinal nerve fiber thickness (RNFL) of 105 *μ*m OD and 191 *μ*m OS. OCT of the optic nerve head showed bilateral dense vitreous adhesions to the optic disc nasally causing traction and optic nerve head elevation of the left eye (Figure 3a,b).

**Figure 4:**
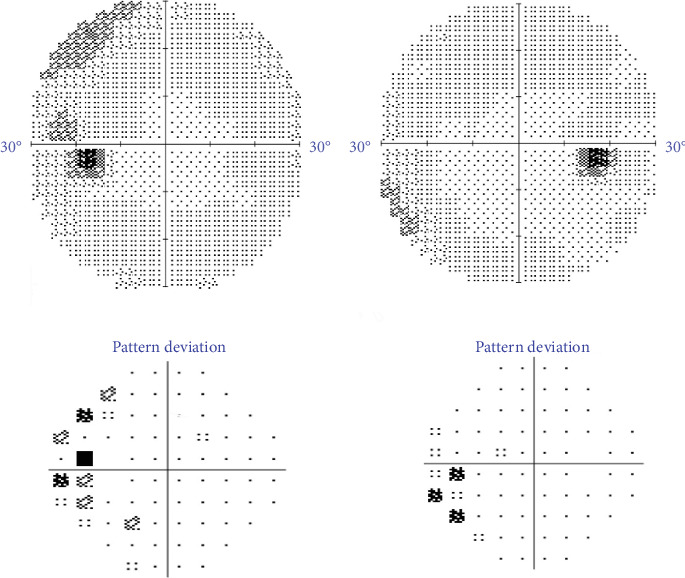
Humphrey visual testing showing improvement of the visual field defect of the left eye.

This initiated further work-up that included magnetic resonance imaging (MRI) of the brain and orbits, erythrocyte sedimentation rate (ESR), and C-reactive protein (CRP). MRI did not reveal any intracranial lesion or suggestive signs of increased intracranial pressure (ICP), and similarly, ESR and CRP were within normal limits. The patient was diagnosed with bilateral VPT causing optic nerve head elevation OS, and observation was recommended. Four months after the initial visit, the patient reported improvement of his visual complaint OS with resolution of optic nerve elevation and repeated OCT showed decreased average RNFL thickness to 99 *μ*m OD and 96 *μ*m OS. Additionally, an improvement of the visual field defect in the left eye was seen on repeated testing (Figure 4).

## 3. Discussion

The vitreous body is a gel-like substance which occupies the space between the lens and the retina. It is composed predominantly of water, along with a small fraction of lipids, collagen, and hyaluronic acid [3]. The optic nerve head is the region where the optic nerve exits the eye, transmitting visual information to the brain. As individuals age, the vitreous body undergoes liquefaction which can cause PVD, a separation of the vitreous cortex from the internal limiting membrane. However, in some cases, the vitreous remains abnormally adhered to the optic nerve head which creates a tractional force termed VPT. Persistent attachment can lead to mechanical pulling on the optic disc, resulting in optic nerve head elevation and various retinal changes. This adherence can be caused by a primary anomaly or by diseases that lead to cellular proliferation [2]. One such anomaly was reported in a case report that described a man in his mid-20s who presented with decreased vision in his left eye. Further investigation revealed persistence of the hyaloid artery, a rare fetal remnant, causing vitreomacular traction and VPT [4]. The clinical presentation of VPT can be quite variable, contributing to its frequent misdiagnosis. Patients with VPT can be asymptomatic or complain of cloudy vision, as noted in our patient, and/or visual field defects. Occasionally, cloudy vision or visual field defects can be preceded by photopsia, which are brief, sudden, flash-like lights. In most of VPT cases, the symptoms are temporary and often resolve on their own [2].

A key feature of VPT is optic nerve head elevation. This was described in a case report that identified VPT as a potential cause of unilateral optic nerve head elevation in two elderly patients. Detailed ocular examinations and B-scan ultrasonography confirmed that persistent traction from the posterior hyaloid on the optic nerve head can simulate optic nerve head elevation and optic disc edema [5]. An important note for clinicians to keep in mind is the potential to mistake bilateral optic nerve head elevation with papilledema. One case report described a 47-year-old woman that presented with bilateral optic disc swelling and blurry vision who was initially misdiagnosed with pseudotumor cerebri. OCT later revealed bilateral VPT, highlighting its potential to mimic papilledema [6]. Another key complication of VPT is intrapapillary and peripapillary hemorrhage due to mechanical stress exerted by the vitreous on the peripapillary capillaries. While these hemorrhages are typically small and self-limiting, their presence can add to the diagnostic challenge, as they may suggest other vascular or inflammatory optic nerve pathologies. One study described eight patients, predominantly Asian Americans, aged 11–42 years old with VPT-related disc hemorrhages. The hemorrhages were mostly found in the superior half of the disc, associated with partial PVD. They resolved over months without visual impairment, emphasizing the need to recognize VPT as a potential cause of disc hemorrhages, especially in younger patients with myopia [7].

VPT has been found to be associated with both diabetic macular edema and diabetic retinopathy. One study described 10 patients with diabetic macular edema, nine of which were refractory to argon laser photocoagulation, who were found to also have optic nerve head elevation. OCT findings confirmed VPT in all 10 patients and parapapillary serous retinal detachment in two eyes. However, it is unknown as to whether the VPT was present prior or after laser treatment [8]. Similarly, VPT has also been associated with diabetic retinopathy. In diabetic retinopathy, the abnormal metabolic environment and elevated glucose levels affect vitreous proteins causing changes in the vitreous, making it more adherent to the optic nerve head. This increased adhesion can result in vitreopapillary tractional forces that distort the optic nerve head and its vasculature, potentially leading to optic nerve dysfunction and atrophy [9]. The tractional force exerted can lead to elongation and thinning of optic nerve fibers, potentially affecting axoplasmic flow and blood flow to the optic nerve head. Specifically, these tractional forces are more commonly located on the nasal side. While observation is typically the first-line management for patients with VPT, surgical intervention has shown success when treating VPT associated with diabetic retinopathy [9]. A study involving 17 patients with diabetic retinopathy and VPT found that surgical intervention via pars plana vitrectomy resulted in improved visual acuity in most patients postoperatively. Additionally, there was a reduction in latency and improvement in visual evoked potentials after surgery in many patients, indicating improved optic nerve function [10].

VPT has also been found to be associated with CRVO. One study documented the presence of VPT, as demonstrated by OCT, in three patients with ischemic CRVO. VPT was detected several months following the CRVO event even after undergoing surgical treatment, suggesting it may be a natural complication of ischemic CRVO. They found two patterns of traction on OCT: eccentric traction on the disc margin and central, symmetric traction. These findings suggest that the management of CRVO should take into consideration the potential sequelae of VPT. Surgical management may be necessary to remove the posterior hyaloid to address the traction forces effectively. Early diagnosis of VPT using OCT is crucial in patients with ischemic CRVO, as delays in surgical intervention can lead to irreversible retinal or macular detachment and atrophy. Therefore, recognizing the mechanisms and implications of VPT can guide more effective surgical interventions and improve prognosis for patients with ischemic CRVO [11].

VPT has also been proposed to be a cause of NAION. This is primarily based on the hypothesis that VPT can lead to dynamic stretch injury, potentially resulting in axonal cytoskeleton fractures and membrane disruptions along with the fact that VPT can cause optic neuropathy symptoms, such as an afferent pupillary defect [12]. One study even demonstrated significant visual acuity improvement in NAION patients undergoing pars plana vitrectomy for presumed VPT, indicating a possible association [13]. However, OCT studies have found no evidence of VPT in patients with NAION [12]. Furthermore, patients with asymptomatic optic disc edema do not progress to optic neuropathy in most cases, indicating that VPT does not necessarily lead to NAION.

Historically, diagnosing VPT and optic nerve head elevation was very difficult, often requiring extensive, expensive evaluations and referrals to neuro-ophthalmic and retina specialists. However, OCT has shown to be a valuable tool in confirming the diagnosis of VPT. It provides high-resolution cross-sectional images of the retina and optic nerve head, allowing clinicians to visualize the vitreous traction and its effects on the optic disc. The characteristic finding in VPT is an elevated optic nerve head with attached vitreous strands extending to the peripapillary region. OCT can also be helpful in differentiating between VPT and other conditions, especially those that may mimic optic disc swelling, such as bilateral papilledema [2], or those that mimic VPT symptomology, such as optic neuropathy [14, 15]. Previous studies reported characteristic OCT findings of temporal wedge visual defects in patients with VPT, as was seen in our patient [15]. Clinicians should be aware of VPT as a cause for optic disc elevation, and OCT of the optic nerve head in suspected cases is crucial for proper diagnosis and management. Observation is usually recommended as the first step of management; however, surgical release of VPT by pars plana vitrectomy might be required in nonresolving cases [16, 17].

## 4. Conclusions

In conclusion, VPT is a notable condition that can lead to optic nerve head elevation, blurring of the disc margins, peripapillary hemorrhages, and other visual disturbances. We report a 64-year-old male who presented with blurred vision and was diagnosed with VPT OCT, which revealed bilateral vitreous adhesions causing optic nerve head elevation in the left eye. Despite the initial visual impairment, the patient's condition improved over time with conservative management. This case highlights the importance of OCT in diagnosing VPT and distinguishing it from other conditions such as papilledema and optic neuropathy. Early recognition and appropriate monitoring are essential for managing VPT effectively, and surgical intervention may be necessary in persistent cases.

## Data Availability

The data that support the findings of this study are available from the corresponding author upon reasonable request.
